# Identification and functional characterization of the distinct plant pectin esterases PAE8 and PAE9 and their deletion mutants

**DOI:** 10.1007/s00425-014-2139-6

**Published:** 2014-08-13

**Authors:** Amancio de Souza, Philip A. Hull, Sascha Gille, Markus Pauly

**Affiliations:** 1 Department of Plant and Microbial Biology, Energy Biosciences Institute, University of California, Energy Biosciences Building 212C, 2151 Berkeley Way, Berkeley, CA 94720-5230 USA; 2 Gladstone Institute of Virology and Immunology, PO Box 419100, San Francisco, CA 94141-9100 USA; 3 Bayer CropScience, Weed Control Biochemistry and Biotechnology, 65929 Frankfurt am Main, Germany

**Keywords:** Acetylesterase, Acetylation, Apoplast, RGI, Cell wall

## Abstract

**PAE8 and PAE9 have pectin acetylesterase activity and together remove one-third of the cell wall acetate associated with pectin formation in Arabidopsis leaves. In** ***pae8*** **and** ***pae9*** **mutants, substantial amounts of acetate accumulate in cell walls. In addition, the inflorescence stem height is decreased.**

Pectic polysaccharides constitute a significant part of the primary cell walls in dicotyledonous angiosperms. This diverse group of polysaccharides has been implicated in several physiological processes including cell-to-cell adhesion and pathogenesis. Several pectic polysaccharides contain acetyl-moieties directly affecting their physical properties such as gelling capacity, an important trait for the food industry. In order to gain further insight into the biological role of pectin acetylation, a reverse genetics approach was used to investigate the function of genes that are members of the Pectin AcetylEsterase gene family (PAE) in Arabidopsis. Mutations in two members of the PAE family (*PAE8* and *PAE9*) lead to cell walls with an approximately 20 % increase in acetate content. High-molecular-weight fractions enriched in pectic rhamnogalacturonan I (RGI) extracted from the mutants had increased acetate content. In addition, the *pae8* mutant displayed increased acetate content also in low-molecular-weight pectic fractions. The *pae8/pae9*-*2* double mutant exhibited an additive effect by increasing wall acetate content by up to 37 %, suggesting that the two genes are not redundant and act on acetyl-substituents of different pectic domains. The *pae8* and *pae8*/*pae9*-*2* mutants exhibit reduced inflorescence growth underscoring the role of pectic acetylation in plant development. When heterologously expressed and purified, both gene products were shown to release acetate from the corresponding mutant pectic fractions in vitro. PAEs play a significant role in modulating the acetylation state of pectic polymers in the wall, highlighting the importance of apoplastic metabolism for the plant cell and plant growth.

**Electronic supplementary material:**

The online version of this article (doi:10.1007/s00425-014-2139-6) contains supplementary material, which is available to authorized users.

## Introduction

Pectins are one of the major components of the primary cell walls of dicots, representing approximately 35 % of the dry mass in these cellular structures (Carpita and Gibeaut 1993; Mohnen 2008). This highly diverse group of polysaccharides has been implicated in cellular processes including cell adhesion, cell growth and pathogen perception (Anderson et al. 2012; McNeil et al. 1984; Ridley et al. 2001). Pectins are divided into four structurally distinct groups: homogalacturonan (HG), rhamnogalacturonan I (RGI), rhamnogalacturonan II and xylogalacturonan (Harholt et al. 2010). The common feature amongst these four groups is the presence of galacturonic acid in the backbone of these polymers. Homogalacturonan is the most abundant pectin polymer in the dicot wall. It consists of a backbone of α-1,4-linked galacturonic acid that can be methyl esterified at the C-6 position (Atmodjo et al. 2013). HG contributes to the general cohesiveness of the cell wall via pectic cross-linking by calcium bridges (Albersheim et al. 2011; McCann and Roberts 1996; Ralet et al. 2008). RGI is the second most abundant pectic polymer in the primary wall. It consists of a repeating disaccharide of *α*-1,4-d-GalA-*α*-1,2-l-Rha. The rhamnosyl-residue can be further substituted with side chains composed of arabinose and galactose, generating large branched structures (Atmodjo et al. 2013; Ralet et al. 2005). The biological role of RGI in the cell wall is still not clear; however, the available data suggest that it plays a structural role in wall architecture (Harholt et al. 2010; Jones et al. 2003; Ulvskov et al. 2005).

All pectic polysaccharides are to a certain extent *O*-acetylated (Harholt et al. 2010; Ralet et al. 2005). The acetyl-moieties are usually attached to the O-2 and O-3 positions of the galacturonic acid moiety of both HG and RGI (Ishii 1997; Keenan et al. 1985). Acetylation of pectins impacts the gelation properties of this class of polymers, which is relevant for the food industry (Ralet et al. 2003). The acetate groups could negatively interfere with the calcium cross-linking between HG polymers via steric hindrance (Ralet et al. 2003).

The degree of pectin acetylation can be modulated by two processes: addition of acetyl-substituents in the Golgi apparatus, the site of pectin synthesis and/or removal of acetyl-substituents by acetylesterases after deposition in the apoplast.

Biochemically, it has been demonstrated that acetyl-CoA provides the substrate for *O*-acetylation in the Golgi (Pauly and Scheller 2000). Recently, several proteins involved in the transfer of acetate to wall polymers have been identified. One of these proteins is reduced wall acetylation (RWA), a putative acetyl-substrate transporter. When knocked out, the acetylation of multiple wall polymers is affected including pectic polysaccharides (Manabe et al. 2011, 2013). Another class of proteins that impact polymer *O*-acetylation are the members of the TBL gene family, which represent putative polymer specific acetyltransferases as demonstrated by TBL27/AXY4 or TBL29 involved in xyloglucan *O*-acetylation (Gille et al. 2011b) or xylan *O*-acetylation, respectively (Xiong et al. 2013). A pectin acetyltransferase has not been identified to date, but it is likely that such a gene is also a member of the TBL family (Gille and Pauly 2012).

Pectin acetylesterases have first been characterized by in vitro activities of purified proteins (Bordenave et al. 1995; Breton et al. 1996; Christensen et al. 1996; Williamson 1991). The identified plant pectin acetylesterases are members of the carbohydrate esterase family 13 [CE13; (Henrissat et al. 2001)]. Members of CE13 are able to hydrolyze acetylester bonds from pectins, but the specific substrate recognized by these enzymes has yet to be demonstrated (Bordenave et al. 1995; Christensen et al. 1996; Gou et al. 2012; Orfila et al. 2012; Williamson 1991). When a poplar pectin acetylesterase was overexpressed in tobacco, the transgenic plants displayed aberrant floral structures and severely collapsed pollen grain walls demonstrating an impact of pectin acetylation on plant tissue morphology and plasticity (Gou et al. 2012). The expression of a mung bean pectin acetylesterase in potato plants resulted in tubers with stiffer and stronger wall matrixes (Orfila et al. 2012). The overexpression of fungal pectin acetylesterases in Arabidopsis resulted in the up-regulation of genes involved in wall polymer *O*-acetylation (*RWA*s) and resistance to specific fungal pathogens (Pogorelko et al. 2013). As these few examples demonstrate, the function of *O*-acetyl groups in the life cycle of plants is beginning to emerge. In this study, we aim at gaining more insights by studying putative pectin acetylesterases in Arabidopsis.

## Materials and methods

### Plant growth

*Arabidopsis thaliana* ecotype Col-0 seeds were stratified at 4 °C for 24 h in a 0.15 % agar solution and pipetted onto soil. Pots were placed in chambers maintained at long-day conditions (16 h light/8 h dark) with a 170–190 μmol m^−2^ s^−1^ light intensity at 22 °C. Plant tissue was harvested at 35 days. At that time also stem height measurements were made from the base of the rosettes to the highest flower in the main inflorescence. *Nicotiana benthamiana* plants used for transient gene expression were grown for 2 weeks, then transplanted into destination pots and grown for an additional 4 weeks until use. These plants were grown under long-day conditions (16 h light/8 h dark) with 170–190 μmol m^−2^ s^−1^ light intensity at 26 °C. All plants were fertilized once with Miracle Grow All-purpose Plant Food (Scotts) according to manufacturer’s recommendation and watered as needed.

### PAE vector constructs

For cloning purposes DNA amplification fragments were generated using Phusion DNA Polymerase (Finnzymes). The putative native promoter and genomic sequence for At4g19420 were cloned into pCR™8/GW/TOPO (Invitrogen) and later introduced via a *Sma*I/*Sac*I restriction/ligation into pPZP221 (Hajdukiewicz et al. 1994) for complementation. For the overexpression of PAE9 in the *pae9*-*1* background, Gateway^®^ Cloning was used. The PAE9 (At5g23870.1) coding sequence (cds) was amplified from leaf cDNA with attB sites containing primers (Table S1) and recombined in a BP reaction with the pDONOR221 vector (Invitrogen). This vector was then recombined in an LR reaction with the binary vector pORE E4 (Coutu et al. 2007) containing the enTCUP2 promoter for overexpression (Schultink et al. 2013). The PAE9 cds was amplified from a vector (pDONOR221 containing AT5G23870.1) and tagged with 6X His at its C-terminus. This fragment was cloned into the pCR™8/GW/TOPO vector backbone. Conventional cloning, using restriction digests (*Xho*I/*BamH*I) and ligation, was used to clone the PAE9 cds tagged with 6X His into pART7 (Gleave 1992) followed by cloning into the pART27 binary vector (Gleave 1992). The PAE8 cds was amplified from Arabidopsis cDNA and Gibson cloning (Gibson et al. 2009) was used to generate PAE8cds:6XHIS in pART7 followed by conventional cloning into pART27 for protein expression in tobacco. The New England Biolabs Gibson Assembly cloning kit was used according to manufacturer’s recommendation as well as the on-line tool NEBuilder™. Binary vectors were transformed into the *Agrobacterium tumefaciens* strain GV3101 which was then used to dip transform (Clough and Bent 1998) *A. thaliana* (*pae8*, SALK_132026; *pae9*-*1*, SALK_046973C) or infiltrate *N. benthamiana*.

### Protein expression, extraction, purification and western blots

For transient protein expression in tobacco, the agrobacterium infiltration buffer used contained 10 mM MES, pH 5.6, 10 mM MgCl_2_ and 0.15 mM acetoseryngone. The cells were suspended in OD_600_ 1.4–2.0 and incubated for 3–4 h with acetoseryngone. Plants were then immersed in infiltration buffer containing cells and vacuum infiltrated 3 times for 3 min each. A construct overexpressing the P19 gene silencing suppressor (Voinnet et al. 2003) was always infiltrated in conjunction with the construct of interest (PAE8:6XHIS or PAE9:6XHIS in pART27) or the empty vector (pART27; final OD_600_ ratio of 1:0.7, respectively).

To identify and verify enzymatic activity of proteins transiently expressed in *N. benthamiana* proteins were extracted from pre-ground tobacco leaves (mortar and pestle in liquid nitrogen). The powder of pre-ground leaves (equivalent to ~1 mL in 2 mL Eppendorf tubes) was ground again in a Retsch ball mill (25 Hz, 2.5 min; company) and extraction buffer added (1 M NaCl; 50 mM Na_2_PO_4_, pH 8; 10 mM imidazole; 1X Halt™ Protease Inhibitor, Thermo Scientific 1861278; 2 mM β-mercaptoethanol). The suspension was incubated under gentle agitation at 4 °C for 1 h, spun down at 20,800 g for 10 min and supernatant collected for protein purification. Protein content of supernatant was measured using the Bradford assay (Bio-Rad protein assay) to normalize protein concentration for Ni–NTA bead (Qiagen 1018240) loading (20 µL of resin/2 mL protein extract). Beads were incubated with protein for 1 h at 4 °C under gentle agitation and collected into a mini spin column (Pharmacia Biotech) after a 500 g, 1 min spin down. The beads were washed 5 times with 250 µL extraction buffer, 4 times with 250 µL washing buffer (300 mM NaCl, 50 mM Na_2_PO_4_ pH 8, 20 mM imidazole) and 6 times with 50 µL elution buffer (300 mM NaCl, 50 mM Na_2_PO_4_ pH 8, 150 mM imidazole). The eluate was buffer exchanged with 50 mM amonium formate, pH 4.5 in a 500 µL Vivaspin column (MWCO of 5,000 Da, Sartorius Stedim Biotech). A final volume of ~200 µL was recovered, which was used for activity assays and western blots.

After proteins were obtained and denatured in loading buffer (NuPAGE LDS Sample Buffer 4X, Invitrogen, NP0007) at 70 °C for 10 min, 20 µL of protein was loaded onto an SDS polyacrylamide gel (10 % Criterion™ Precast Gel). These were then wet blotted onto nitrocellulose membranes using transfer buffer [0.075 % (v/v) ethanolamine, 0.0935 % (w/v) Glycine and 20 % (v/v) ethanol] at 100 V for 80 min at 4 °C. The membrane was then blocked overnight at 4 °C in 50 mM Tris Hcl, 150 mM NaCl and 0.5 % Tween (TBS-T) containing 3 % (w/v) nonfat powdered milk. Primary antibody (mouse anti 6X HIS, Fisher 50272472) was added (1:3,000, v/v) and incubated for 3 h at room temperature. This was followed by three 10-min washes with TBS-T and incubation in TBS-T containing 3 % (w/v) nonfat powdered milk with the secondary HRP conjugated antibody (Goat Anti-mouse IgG HRP Conjugate, Invitrogen, M30102; 1:3,000, v/v) for 1 h at room temperature. After another series of TBS-T washes (3) the membrane was developed by adding chemiluminescent reagents (Genscript LumiSensor) and visualized using the Fuji LAS-4000 imager.

### Protein sequence analysis

Protein sequence alignments and trees were generated using the Seaview4 and the MUSCLE software packages.

### Plant cell wall preparations

Stems tissue (15–20 mg of stem lower internode) was pre-ground in large metal ball containers (Retsch MM 400; 30 Hz for 30 s). For stem preparations and the reverse genetics screen (Table 1; leaves) dry plant material (20–40 mg of leaf or 10–20 mg of stem material) was ground in liquid nitrogen using 3 small metal balls in 2 mL plastic tubes twice (Retsch MM 400; 25 Hz for 2.5 min). The ground material was washed twice with 70 % ethanol (1.5 mL) by vortexing, pelleting the wall material (20,800 g for 10 min) and discarding the supernatant. The residue was washed three times with a 1:1 (v:v) methanol:chloroform solution (1.5 mL) using the same pelleting conditions as described above. The pellet was dried in a speed vacuum centrifuge at 60 °C for 15 min (Eppendorf Vacufuge™).

**Table 1:** Arabidopsis lines investigated in this study

Gene	Mutant line name	Insertion line	RT-PCR^a^	Leaf acetate content (%)^b^	Standard deviation (%)
At2g46930	*pae3*-*1*	SALK_066524C	ND	103.6	3.1
	*pae3*-*2*	SALK_137505C	K-down	106.5*	3.0
At3g09410	*pae5*-*1*	SALK_140555	ND	104.4	4.9
	*pae5*-*2*	SALK_052303C	K-down	104.0	2.8
At3g62060	*pae6*-*1*	SALK_020618	WT	106.2*	0.8
	*pae6*-*2*	SALK_134907	ND	105.0	5.4
At4g19410	*pae7*-*1*	SALK_093502C	WT	113.3*	1.3
	*pae7*-*2*	GABI_272B08	K-down	106.5*	1.4
At4g19420	*pae8*	SALK_132026	ND	118.2*	4.4
At5g23870	*pae9*-*1*	SALK_046973C	ND	120.3*	1.3
	*pae9*-*2*	GABI_803G08	ND	121.6*	2.9
At5g26670	*pae10*-*1*	SALK_043807	ND	94.1	11.7
	*pae10*-*2*	SAIL_802_C05	ND	104.1	1.2
At5g45280	*pae11*-*1*	SALK_049340.48.65.x	ND	123.05	3.1
	*pae11*-*2*	GABI_505H02	ND	135.0*	3.8
At3g05910	*pae12*-*1*	GABI_018A02	ND	100.4	9.7
	*pae12*-*2*	GABI_646F06	ND	105.7	2.0

* Significant differences based on *t* test (*P* < 0.05); *n* ≥ 4

^a^RT-PCR results are categorized as transcript not detected (ND), knockdown (K-down) or WT-level (WT)

^b^Leaf acetate content of 5- to 6-week-old plants was normalized to WT (WT 100 %)

Plant cell wall preparations for *PAE8* and *PAE9* lines used for pectin digest, substrate production and size exclusion chromatography (SEC) were conducted in larger volumes. Up to 600 mg of dried 35-day-old leaf material was ground (200 mg at a time) in large metal ball mill grinders (Retsch ball mill MM 400) two times at 30 Hz for 30 s. This procedure produced a fine powder, 500 mg of which was washed 4 times with 70 % ethanol (30 mL per wash) by vortexing and pelleting of the wall material (3,220 g for 10 min). The supernatant was discarded and the pellet was washed 4 times with a 1:1 methanol:chloroform solution (30 mL per wash) using the same pelleting conditions as described above. The pellet was dried at room temperature for 48 h followed by at least 1 h in the lyophilizer.

### Enzymatic de-starching and pectinase digest

Starch was removed from the small cell wall preparations (Table 1) by enzymatic digestion. A reaction consisted of 13–19 mg wall material in 1 mL McIlvane buffer, pH 5. The suspension was vortexed and incubated at 80 °C for 20 min and then cooled on ice. A reaction buffer was added to a final concentration of 0.065 μg mL^−1^ sodium azide; 0.65 μg mL^−1^ α-amylase (Sigma A-6380); 12.8 U mL^−1^ of Pullulanase M2 (Megazyme). The digest was incubated at 37 °C at 230 rpm for 15 h. The digest was stopped by incubation at 99 °C for 10 min. Wall material was pelleted by centrifugation (3,220*g* for 10 min) and the supernatant discarded. The pellet was washed with equal volumes of water (30 mL) for 3 times. Finally, a 70 % aqueous ethanol wash of equal volume was performed before the pellet was dried at room temperature followed by vacuum treatment (Eppendorf Vacufuge™).

For larger cell wall preparations the de-starching digest included 150–170 mg wall material in McIlvane buffer, pH 5 (~13 mg mL^−1^). The suspension was vortexed and incubated at 80 °C for 20 min and then cooled on ice. A reaction buffer was added with a final concentration of 0.065 μg mL^−1^ sodium azide; 0.65 μg mL^−1^ α-amylase (Sigma A-6380); and 12.8 U mL^−1^ of Pullulanase M2 (Megazyme). The digest was incubated at 37 °C at 230 rpm for 15 h. The digest was stopped by incubation at 80 °C for 20 min. Wall material was pelleted by centrifugation (3,220*g* for 10 min) and the supernatant discarded. The pellet was washed with equal volumes of water (30 mL) for 3 times. Finally, a 70 % aqueous ethanol wash of equal volume was performed before the pellet was dried at room temperature followed by vacuum treatment (Eppendorf Vacufuge™).

De-starched wall material was treated with pectinases to generate the pectic extract. The enzymatic reaction (per mL) was conducted with 6 mg of material in 50 mM ammonium formate, pH 4.5, containing 0.2 μg sodium azide, 2 mU of endopolygalacturononase M2 (EC3.2.1.15; Megazyme) and 0.04 mU of pectin methyl esterase (EC3.1.1.11; Novozymes, Christgau et al. 1996). The digest was incubated for 17.5 h at 37 °C with 230 rpm agitation and stopped by incubation at 80 °C for 20 min. The pellet was spun down (3,220 g for 10 min) and the supernatant, representing the pectic extract, was filtered through a 0.45-μm syringe filter (mini sart high flow 16533, Satorius Stedin). The pellet was washed 3 times with 15 mL water before drying.

### Pectin fractionation using SEC

The pectic extract was freeze-dried and resuspended in 50 mM amonium formate, pH 4.5 (1 mL). The concentrated pectic extract was subjected to size exclusion chromatography using a superpose 12 10/300 GL column (Amersham Biosciences). The column was connected to an Akta Purifier FPLC (General Electric) or a PL-GPC 50 (Varian Inc.) chromatographer. The column was equilibrated in 50 mM amonium formate, pH 4.5, using a 0.4-mL min^−1^ flow. Samples were injected manually using a 100-μL loop. Fractions were collected in 30-s intervals in a 96-well microtiter plate (Greiner bio-one) and lyophilized. The resulting pectic fractions were resuspended in water (100 μL) and used for subsequent analysis. Dextran standards of known sizes were used to calibrate the column.

### Acetic acid measurements of de-starched walls, pectic extract and pectin fractions

Wall preparations (10 mg/mL), pectic extract or pectic fractions were saponified by adding an equal volume of 1 M NaOH and incubation for 1 h at 26 °C, under gentle agitation of 600 rpm. The de-esterified samples were neutralized with an equal volume of 1 M HCl. The reaction was pelleted for 10 min at 20,800 *g* and the acetic acid content of the supernatant (10–50 μL) determined using the Acetic Acid Kit (K-Acet, Megazyme) as described (Gille et al. 2011a).

### Activity assays

Recombinant protein derived from tobacco leaves (PAE9 and PAE8) was tested for activity against pectin fractions. The added protein was equalized based on protein content and was incubated for 18 h with 40 μL of each pectic fraction in a total volume of 60 μL (50 mM ammonium formate, pH 4.5, for PAE9 and pH 5.0 for PAE8). After incubation the acetate released was measured using the Acetic Acid Kit (K-ACET, Megazyme).

### Transcriptional analysis of *A. thaliana* (RT-PCR and Q-RT-PCR)

For RT-PCR analysis total RNA was extracted from plant tissue using the Plant RNeasy kit from Qiagen according to manufacturer’s protocol. Total RNA (3.5 µg) was treated with DNAse (Roche 04716728001) in a 20-µL reaction using 1 µL DNAse and 2 µL of 10 X buffer. Treatment was carried out at 37 °C for 15 min and followed by a 70 °C denaturation step for 15 min. The Superscript III First Strand Synthesis kit from Invitrogen was used to synthesize cDNA and non-RT controls from 250 ng of total RNA. The cDNA (2 µL) was then used in JumpStart Red Taq ReadyMix (Sigma) PCR reactions. The polypyrimidine tract-binding protein 1 [PTB, (Gille et al. 2009)] gene was used as an expression control.

For Q-RT-PCR analysis total RNA was extracted from plant tissue using the Plant RNeasy kit from Qiagen according to manufacturer’s protocol. DNase treatment for Q-RT-PCR was performed using the Ambion TURBO DNA-free™ kit. Treated total RNA (200 ng) was then used for cDNA synthesis using the M-MLV Reverse Transcriptase kit (Invitrogen 28025-013) following manufacturer’s instructions. The templates (1 µL) were used for Q-RT-PCR reactions with the Thermo Scientific Maxima SYBR Green/ROX Q-PCR Master Mix. Reactions were set up according to instructions from the manual and Q-RT-PCR reactions were run on a StepOnePlus Real-Time PCR System from applied Biosciences. Cycling conditions commenced with 10 min at 95 °C followed by 40 cycles of 95 °C for 15 s, 60 °C for 30 s and 72 °C for 30 s. Relative abundance of transcripts was calculated by the Applied Biosciences software using the PTB gene as the internal control.

### Monosaccharide analysis using high-performance anion-exchange chromatography with pulse amperometric detection (HPAEC-PAD)

Wall material, pectic extract and remaining residue and pectic fractions were hydrolyzed in 250 μL of 2 M tri-fluoroacetic acid (TFA) at 121 °C for 90 min. The reaction was dried under a flow of nitrogen. The sample was washed two times with 300 µL isopropanol and dried under nitrogen stream each time. The hydrolyzed samples were resuspended in water and subjected to a CarboPac PA20 (neutral sugars) or CarboPac A200 (uronic acids) columns using 3 separate gradient programs to resolve all sugars of interest. For neutral sugar separation a flow rate of 0.4 mL min^−1^ with the following gradient was used: 2 mM NaOH for 20 min; 100 mM NaOH flush for 5 min; and 2 mM NaOH for 5 min for re-equilibration. In parallel a second method was used for neutral sugar separation: 18 mM NaOH for 15 min; 100 mM NaOH flush for 5 min; and 18 mM NaOH for 7 min for re-equilibration. For the separation of uronic acids the following gradient was used: 0.1 M NaOH with a gradient of 50–200 mM sodium acetate from 0 to 10 min; 200 mM NaOH flush for 2 min; 50 mM NaOH for 2.9 min. For quantification purposes standard curves for all measured sugars (fucose, galactose, glucose, xylose, mannose, rhamnose, arabinose, galacturonic acid and glucuronic acid) were used with the following concentrations (μg mL^−1^): 2, 1, 0.5, 0.25, 0.125, 0.0625, 0.03125. To account for possible sugar degradation during the TFA hydrolysis process standard mixtures of known content (6, 3, 1.5, 0.75 and 0.375 μg/individual sugar) of each analyzed sugar were hydrolyzed under the same conditions in parallel with tested samples. Measurements obtained for tested samples were corrected based on measured degradation of standards.

### Statistical analysis

Statistical analysis was performed using one-way ANOVA and Tukey’s test (level of significance: *P* < 0.05; SigmaPlot for Windows Version 11.0) or Student’s *t* test (*P* value as indicated; Microsoft Excel tool).

## Results

### Mutants in the Arabidopsis pectin acetylesterase gene family have walls with increased acetate content and reduced inflorescence stem growth

**Fig. 1:**
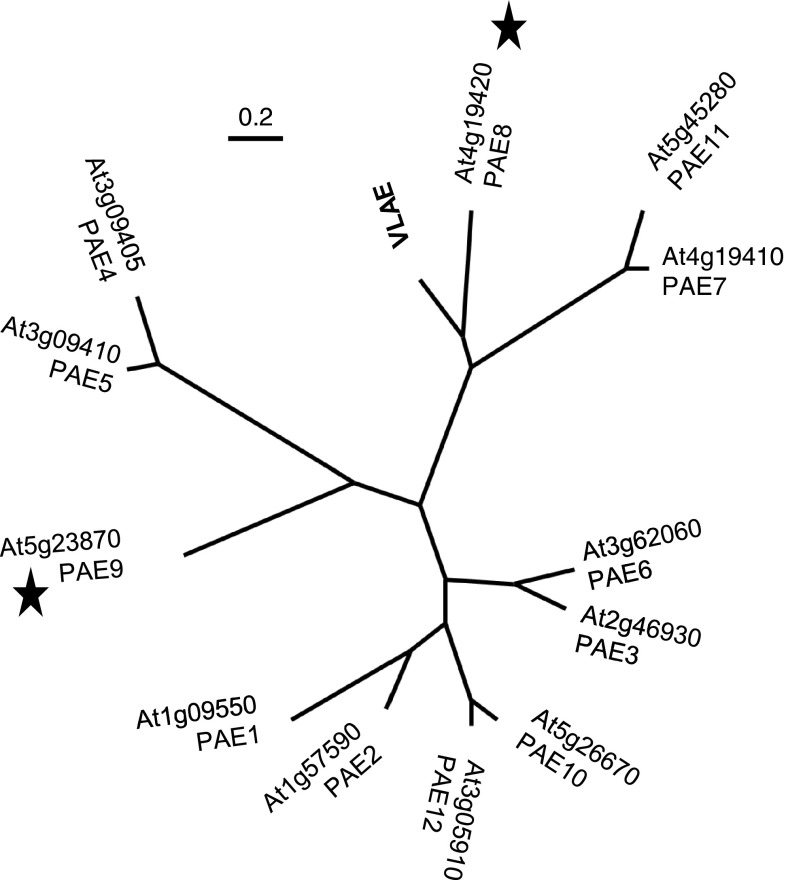
Phylogenetic tree of gene family related to a pectin acetylesterase. Tree constructed using the maximum likelihood method with the Seaview4 software package, which used muscle and PhyML for alignment and tree, respectively. *VLAE* acetylesterase expressed in the developing corm of *Amorphophallus konjac.* *Bar* indicates relative distance between protein sequences. *Black stars* highlight the genes focused on in the present study

**Fig. 2:**
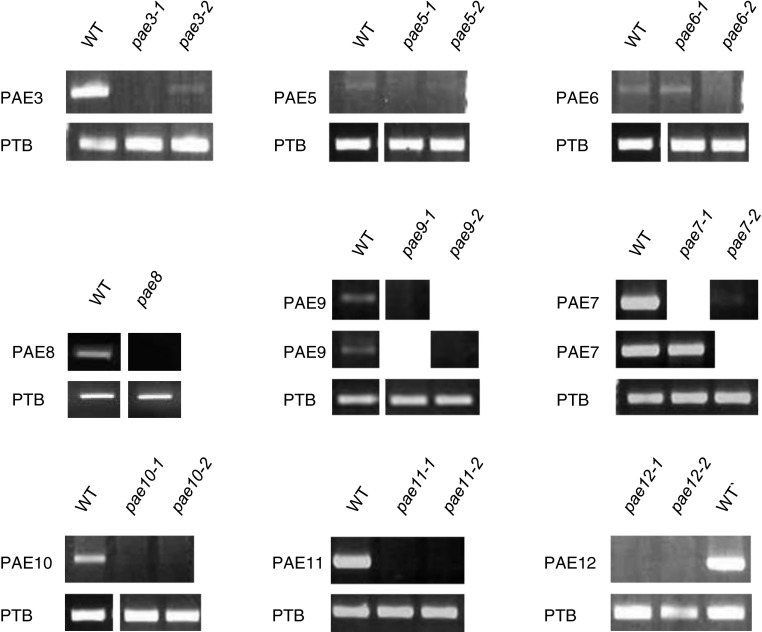
RT-PCR of pectin acetylesterase T-DNA insertion lines. RT-PCR for PAE3 transcript (*pae3*-*1*, SALK_066524C; *pae3*-*2*, SALK_137505C; 3-week-old leaves); PAE5 (*pae5*-*1*, SALK_140555; *pae5*-*2*, SALK_052303C; 3-week-old leaves); PAE6 (*pae6*-*1*, SALK_020618; *pae6*-*2*, SALK_134907; 19-day-old leaves); PAE8 (*pae8*, SALK_132026; 2-week-old leaves); PAE9 (*pae9-1*, SALK_046973C; *pae9-2*, GABI-803G08; 3-week-old leaves); PAE7 (*pae7*-*1*, SALK_093502C; *pae7*-*2*, GABI_272B08, 19-day-old leaves); PAE10 (*pae10*-*1*, SALK_043807; *pae10*-*2*, SAIL_802_C05; 3-week-old leaves); PAE11 (*pae11*-*1*, SALK_049340.48.65.x; *pae11*-*2*, GABI_505H02; 19-day-old leaves); PAE12 (*pae12*-*1*, GABI_018A02; *pae12*-*2*, GABI_646F06; 3-week-old leaves). PTB—housekeeping gene. WT = Col-0 plants of same age as insertion lines being tested. Primers used for each reaction are listed in Suppl. Table S2

The *A. thaliana* genome contains 12 annotated CE13 genes [Fig. 1; CAZY.org; (Henrissat et al. 2001)]. Based on the availability of T-DNA insertion lines with altered gene expression, the function of nine of those genes (At2g46930, At3g09410, At3g62060, At4g19410, At4g19420, At5g23870, At5g26670, At5g45280, At3g05910) was investigated. Gene expression in mutant lines was established by RT-PCR and at least one line per gene showed reduced expression (Table 1; Fig. 2). Since rosette leaves are rich in pectic polymers (Zablackis et al. 1995) this tissue was chosen for biochemical analyses. Wall material of 5- to 6-week-old leaves was prepared and its alkali labile acetate content determined (Table 1). Some T-DNA lines affected in gene expression exhibited a significant alteration/increase in acetate content including *pae3*-*2* (SALK_137505C), *pae7*-*2* (GABI_272B08) *pae8* (At4g19420; SALK_132026), *pae9*-*1* (At5g23870; SALK_046973C), *pae9*-*2* (At5g23870; GABI_803G08) and *pae11*-*2* (GABI_505H02). PAEs, whose second allel mutants represent a knock-down rather than a knockout allel (PAE3; PAE5; PAE7), or where one or both of the allels did not display a statistically significant increase in acetate content (PAE6, PAE10, PAE11, PAE12) were due to their genetic inconsistencies not further pursued here. Should more knock-out lines become available in the future many of those PAE would certainly be of interest for a follow-up.

**Fig. 3:**
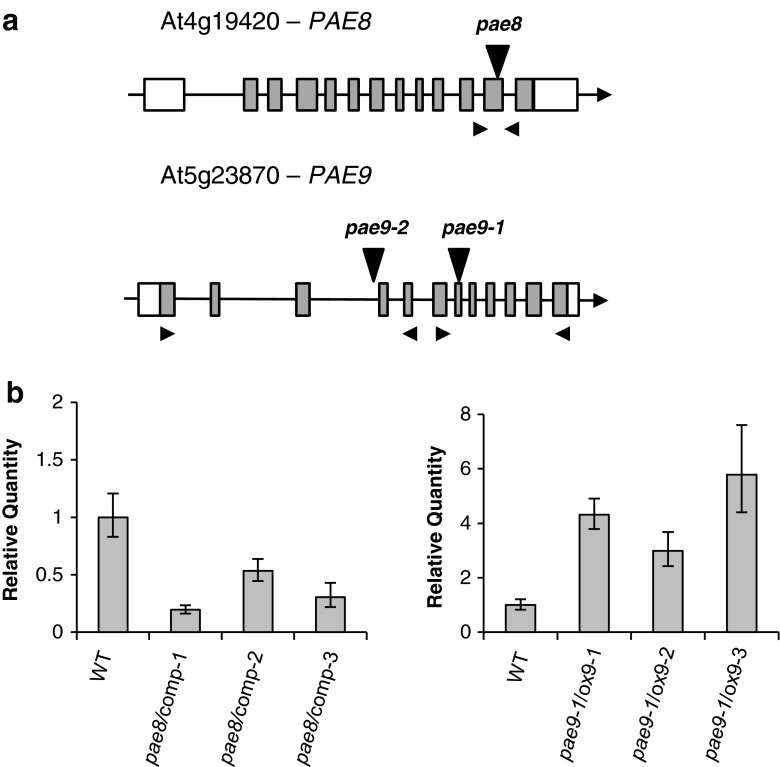
Gene models for *PAE8* and *PAE9* and Q-RT-PCR of complementation and overexpression lines. **a** Gene model for *PAE8* and *PAE9;* *boxes* indicate exons (*gray*, translated regions); *black line* in between exons indicate introns. *Vertical black triangles* indicate position of T-DNA insertions in relation to translation start site (*pae8* SALK_132026, +2,046 bp; *pae9*-*1*, SALK_046973C, +2,712 bp; *pae9*-*2*, GABI-803G08, +1,949 bp). *Small black triangles* indicate primer positions used for RT-PCR. **b** Relative quantity of the *PAE8/PAE9* transcript determined by Q-RT-PCR in 35-/30-day-old leaves of T3 complementation lines. *Error bars* indicate minimum and maximum variation. *PTB* gene expression was used as an internal control for normalization. WT = Col-0 35/30-day-old leaves. *n* = 6

**Fig. 4:**
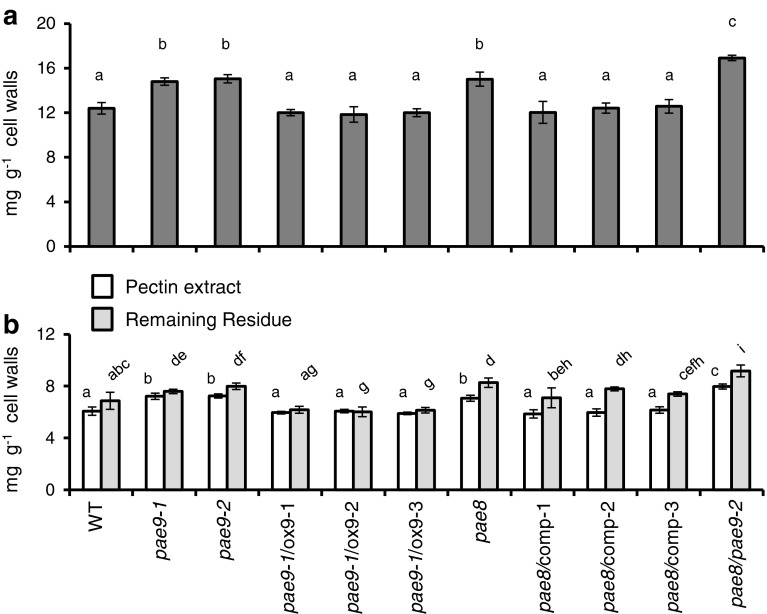
Acetate content of wall (**a**), pectin extract, and remaining residue (**b**) of PAE mutant lines. *Error bars* indicate standard deviation; *letters* indicate statistical significant differences based on one-way ANOVA (*P* < 0.05); *n* ≥ 4

Two knockout alleles of *PAE9* were available (Figs. 2, 3a). When leaf material was tested for altered wall acetate content an approximate 20 % increase was found in both mutant alleles (Fig. 4a). Three independent *PAE9* overexpression lines in the *pae9*-*1* background revealed complementation of the wall acetate back to WT levels. The overexpression lines were shown to have at least 3 times higher transcript levels than WT plants (Fig. 3b). These results indicate that PAE9 is also responsible for modulating leaf wall acetate levels.

Since only one *pae8* knock-out allel was available (Table 1; Fig. 3a), genetic complementation of this *pae8* mutant was performed by transforming the genomic WT *PAE8* sequence under the control of the native *PAE8* promoter into the mutant. Three independently obtained T3 complementation lines were subjected to Q-RT-PCR analysis confirming that the PAE transcript was expressed (Fig. 3b). The *pae8* mutant exhibited an approximate 20 % acetate increase in its leaf walls compared to WT (Fig. 4a). The complementation lines were shown to rescue the mutant phenotype, i.e. wall acetate levels were reduced down to WT levels (Fig. 4a). These data demonstrate that PAE8 modulates the wall acetate level of leaves.

A double mutant, *pae8/pae9*-*2,* was generated and analysis indicated a 37 % increase in total wall acetate content (Fig. 4a), a near additive level compared to the two single mutants.

**Fig. 5:**
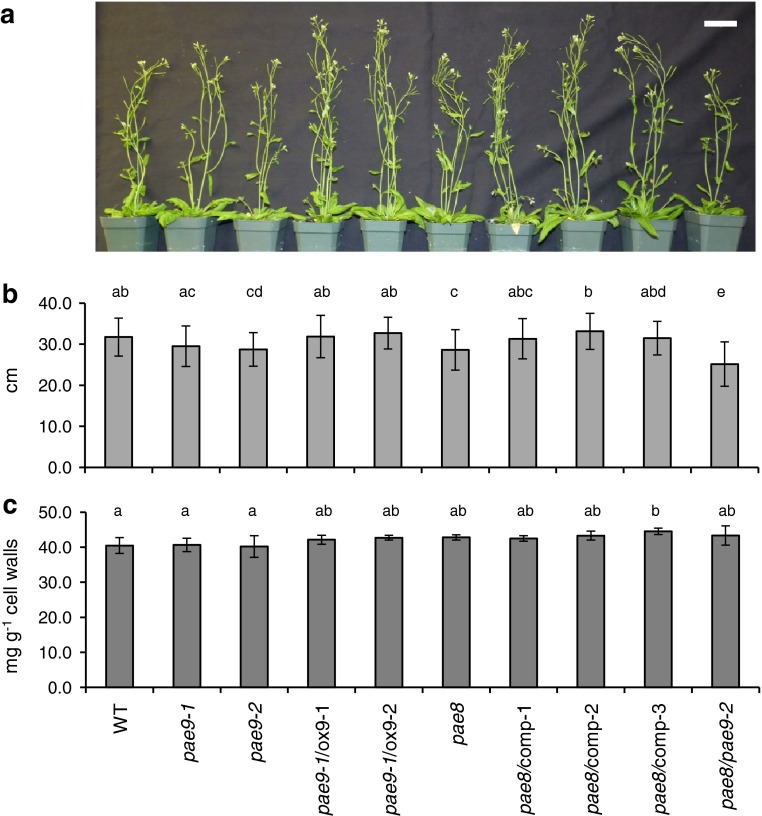
Inflorescence stem heights and stem acetate content. **a** General appearance of 35-day-old Arabidopsis plants; *white bar* 5 cm. **b** Inflorescence height in cm; *n* ≥ 26. **c** Acetate content in the second internode of 35-day-old stems; *n* ≥ 6. *Error bars* indicate standard deviation; *letters* indicate statistical significant differences based on one-way ANOVA (*P* < 0.05)

No morphological changes could be observed in the rosette leaves of these Arabidopsis mutants. However, publically available gene expression data indicated a high expression of both *PAE8* and *PAE9* in the lower internode of Arabidopsis inflorescences (Fig. S1). Indeed, upon examining the inflorescence height of 35-day-old Arabidopsis plants a reduction was observed for *pae8* (10 %; Fig. 5a, b) and the *pae8/pae9*-*2* double mutant (21 %; Fig. 5a, b). The complementation lines for *pae8* restored inflorescence heights to WT levels (Fig. 5b). The double mutant exhibited twice the reduction in height of the one exhibited by the *pae8* single mutant suggesting an additive effect caused by the *pae9*-*2* mutation even though no consistent height reduction was found for the *PAE9* mutants. Due to this developmental alteration acetate content was determined in the stem lower internode of the lines, but no differences in acetate content could be observed between the mutant lines and WT (Fig. 5c).

### The *pae8* and *pae9* mutants have increased pectin acetate content

An attempt was made to identify the wall polymer with enhanced acetyl-substituents in the *pae8* and *pae9* mutants. Since VrPAE1 has been shown to have pectin acetylesterase activity (Bordenave et al. 1995; Breton et al. 1996) the initial investigation focused on the pectic polysaccharides. An enzymatic pectin digest was performed on leaf wall material to extract pectic polymers and its alkali labile acetate content was determined. Indeed, the pectic extracts derived from *pae8*, *pae9*-*1*, *pae9*-*2* and *pae8/pae9*-*2* exhibited an increased acetate content compared to WT (Fig. 4b). In the *PAE8* complementation lines and in the *PAE9* overexpression lines in the *pae9*-*1* background the acetate content was equal to WT levels (Fig. 4b). The monosaccharide composition of the pectic extract of the various mutant lines revealed as expected a dominance in galacturonic acid and rhamnose (Tables 2, 3, Supplemental Fig. S2) indicative of pectins. However, a reduction in the rhamnose content of *pae8* (−12 %) and *pae8/pae9*-*2* (−17 %) in the pectin extracts was observed, but not galacturonic acid, suggesting that RGI in these mutants could be less extractable.

**Table 2:** Rhamnose content of cell wall, pectin extract, residue and fractions in *pae8*, *pae9* and double mutant (mg g^−1^ cell wall)

	WT	*pae9*-*1*	*pae9*-*2*	*pae9*-*1*/ox9-1	*pae9*-*1*/ox9-2	*pae9*-*1*/ox9-3	*pae8*	*pae8*/comp-1	*pae8*/comp-2	*pae8*/comp-3	*pae8*/pae9-2
Cell wall	21.75 ± 1.97	21.44 ± 0.74	21.52 ± 1.18	23.61 ± 0.64	22.97 ± 1.60	22.1 ± 1.05	20.88* ± 1.59	21.46 ± 0.87	21.94 ± 0.55	21.52 ± 3.72	20.51 ± 1.12
Pectin extract	12.56 ± 0.58	12.25 ± 0.37	13.91 ± 3.89	15.23* ± 0.27	14.68* ± 0.47	11.86 ± 2.51	11.04* ± 0.71	11.53 ± 0.72	11.74 ± 0.66	12.1 ± 0.51	10.37* ± 0.60
Pectin residue	9.47 ± 1.63	11.49 ± 0.54	10.86 ± 0.66	10.69 ± 1.55	10.84 ± 1.63	10.58 ± 0.68	9.33 ± 1.47	7.65 ± 0.93	7.79 ± 0.56	8.68 ± 0.15	12.54 ± 1.31
Fraction I	3.20 ± 0.40	3.34 ± 0.19	2.84 ± 0.42	4.00* ± 0.11			2.85 ± 0.34	3.04 ± 0.04			2.65 ± 0.15
Fraction II	2.97 ± 0.66	2.17 ± 0.09	1.93 ± 0.20	3.11 ± 0.18			2.62 ± 0.34	3.74 ± 0.35			1.77* ± 0.19
Fraction III	2.67 ± 0.16	2.65 ± 0.08	2.49 ± 0.20	3.05* ± 0.04			2.54 ± 0.10	2.63 ± 0.14			2.33* ± 0.09
Fraction IV	0.54 ± 0.08	0.57 ± 0.05	0.52 ± 0.01	0.60 ± 0.10			0.50 ± 0.10	0.50 ± 0.05			0.49 ± 0.03
Fraction V	n.d.	n.d.	n.d.	n.d.			n.d.	n.d.			n.d.

The entire monosaccharide composition of these fractions can be found in Figs. S2, S3 and S4

*n.d* not detected, *ox9* overexpression of the *PAE9* coding sequence, *comp* complementation with the native promoter and genomic sequence of *PAE8*, *WT* Col-0

± Indicates standard deviation

* Statistical significant differences from WT based *t* test (*P* < 0.01); *n* ≥ 3

**Table 3:** Galacturonic acid content of cell wall, pectin extract, residue and fractions in *pae8*, *pae9* and double mutant (mg g^−1^ cell wall)

	WT	*pae9*-*1*	*pae9*-*2*	*pae9*-*1*/ox9-1	*pae9*-*1*/ox9-2	*pae9*-*1*/ox9-3	*pae8*	*pae8*/comp-1	*pae8*/comp-2	*pae8*/comp-3	*pae8*/pae9-2
Cell wall	180.4 ± 12.2	171.8 ± 4.0	181.2 ± 3.7	191.2 ± 3.9	176.0 ± 7.5	175.0 ± 9.5	179.7 ± 10.2	175.9 ± 5.1	181.0 ± 5.5	184.7 ± 4.9	169.1 ± 2.6
Pectin extract	192.0 ± 22.9	192.0 ± 0.9	229.2 ± 60.8	205.2 ± 1.0	179.0 ± 11.3	153.4 ± 40.2	171.0 ± 8.5	157.5 ± 14.3	155.6 ± 4.7	172.2 ± 9.5	169.6 ± 6.3
Pectin residue	38.9 ± 6.1	44.0 ± 2.3	44.7 ± 2.6	47.5 ± 3.2	44.4 ± 3.9	42.0 ± 4.7	36.3 ± 5.1	31.3 ± 4.4	32.5 ± 1.2	34.5 ± 1.4	42.7 ± 1.1
Fraction I	5.7 ± 0.7	6.3 ± 0.7	5.3 ± 0.8	7.2* ± 0.3			5.0 ± 0.5	5.3 ± 0.1			4.5 ± 0.4
Fraction II	6.5 ± 0.7	5.7 ± 0.2	5.3* ± 0.3	7.0 ± 1.6			6.2 ± 0.9	6.9 ± 0.7			4.6* ± 0.1
Fraction III	16.6 ± 1.6	16.8 ± 1.0	16.4 ± 1.1	18.0 ± 0.2			16.5 ± 0.6	15.7 ± 0.8			16.4 ± 0.6
Fraction IV	43.0 ± 0.9	41.7 ± 1.9	45.0 ± 4.9	43.7 ± 0.9			44.1 ± 1.9	40.8 ± 2.3			45.3 ± 1.1
Fraction V	65.6 ± 5.9	62.3 ± 11.8	59.0 ± 9.90	67.7 ± 5.4			64.0 ± 4.4	68.3 ± 11.2			56.3 ± 3.2

The entire monosaccharide composition of these fractions can be found in Figs. S2, S3 and S4

*ox9* overexpression of the *PAE9* coding sequence, *comp* complementation with the native promoter and genomic sequence of *PAE8*, *WT* Col-0

± Indicates standard deviation

* Statistical significant differences from WT based *t* test (*P* < 0.01); *n* ≥ 3

The remaining wall residue after pectin extraction was also analyzed for acetate content (Fig. 4b). All remaining residues still contained acetate with changes among the different mutants that were similar to the pectin extracts. The monosaccharide analysis of these residues revealed still the presence of the pectic monosaccharides rhamnose and galacturonic acid demonstrating that the enzymatic pectin extraction conditions used did not lead to the entire solubilisation of pectins and thus removal from the residues (Tables 2, 3; Fig. S2c).

**Fig. 6:**
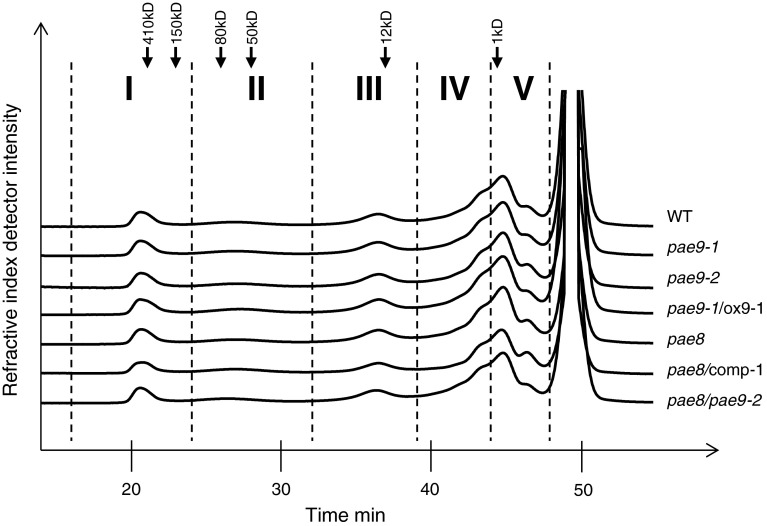
Size exclusion chromatography of pectic extracts. Pectic extracts of PAE mutant lines (*right titles*) were applied to size exclusion chromatography. Fractions were collected according to depicted scheme (*roman numerals*); *dashed bars* indicate fraction borders. *Black arrows* indicate elution time of dextran standards. *ox9* overexpression of the *PAE9* coding sequence. *comp-1* complementation with the native promoter and genomic sequence of *PAE8. n* ≥ 5

The origin of the increase of pectic acetate in the mutants was investigated by fractionation of the pectic extract through size exclusion chromatography (Fig. 6). Distinct peaks were observed for pectic fragments of different molecular weights. The eluted pectic compounds were pooled into five fractions as indicated in Fig. 6. Monosaccharide analysis of the pectin fractions indicates a qualitative separation of pectic domains (Tables 2, 3; Fig. S3; Fig. S4). Fractions I and II contain large molecules (fraction I > 147.5 kDa; fraction II 147.5–23 kDa; Fig. 6), which are enriched for monosaccharides present in RGI structures (galacturonic acid, rhamnose, arabinose and galactose). The ratio between galacturonic acid and rhamnose in fractions I and II is larger than 1 suggesting that these fractions also contain HG. Fraction III (23.2–4.6 kDa) is enriched with galacturonic acid when compared to fractions I and II, however, still containing monosaccharides representative of RGI. In fractions IV (4.6–1.5 kDa) and V (1.5–0.6 kDa) the abundance of galacturonic acid suggests small homogalacturonan fragments. The concentration of sugars in each fraction is also consistent with the chromatogram profiles obtained (Tables 2, 3; Fig. 6; Fig. S3; Fig. S4). Based on the content of acetate per galacturonic acid the degree of acetylation of the polymers in the large-molecular-weight fractions is higher than the lower molecular weight fractions (Tables 3, 4; Fig. S3; Fig. S4). Similar chromatograms (Fig. 6) and monosaccharide content (Tables 2, 3; Fig. S3; Fig. S4) were obtained for the WT and the various mutant lines indicating that no detectable change occurred in the overall pectic polymer size of the single mutants.

**Table 4:** Acetate content of pectic fractions in *pae8*, *pae9* and double mutant (mg g^−1^ cell wall)

	WT	*pae9*-*1*	*pae9*-*2*	*pae9*-*1*/ox9-1	*pae9*-*1*/ox9-2	*pae9*-*1*/ox9-3	*pae8*	*pae8*/comp-1	*pae8*/comp-2	*pae8*/comp-3	*pae8*/pae9-2
Fraction I	1.15 ± 0.08	1.53* ± 0.12	1.45* ± 0.12	1.14 ± 0.05	1.13 ± 0.08	1.09 ± 0.05	1.42* ± 0.09	1.09 ± 0.05	1.13 ± 0.03	1.17 ± 0.07	1.65* ± 0.08
Fraction II	1.43 ± 0.08	1.66* ± 0.04	1.56 ± 0.13	1.35 ± 0.05	1.34 ± 0.05	1.30* ± 0.06	1.68* ± 0.08	1.43 ± 0.08	1.45 ± 0.09	1.52 ± 0.11	1.73* ± 0.09
Fraction III	0.75 ± 0.04	0.81* ± 0.03	0.77 ± 0.05	0.78 ± 0.03	0.78 ± 0.03	0.73 ± 0.05	0.79 ± 0.04	0.71 ± 0.05	0.72 ± 0.03	0.74 ± 0.02	0.81 ± 0.04
Fraction IV	1.20 ± 0.07	1.26 ± 0.06	1.29 ± 0.10	1.15 ± 0.05	1.19 ± 0.06	1.16 ± 0.07	1.43* ± 0.08	1.14 ± 0.08	1.12 ± 0.06	1.19 ± 0.04	1.63* ± 0.08
Fraction V	0.77 ± 0.05	0.78 ± 0.05	0.75 ± 0.06	0.74 ± 0.02	0.73 ± 0.03	0.72 ± 0.06	0.88* ± 0.05	0.77 ± 0.07	0.78 ± 0.04	0.81 ± 0.03	0.91* ± 0.02

*ox9* overexpression of the *PAE9* coding sequence, *comp* complementation with the native promoter and genomic sequence of *PAE8*, *WT* Col-0

± Indicates standard deviation

* Statistical significant differences based *t* test (*P* < 0.01); *n* ≥ 4

In *pae8* all fractions with the exception of fraction III exhibit a significant increase in acetate content (Table 4), while the corresponding complementation lines restored the fraction’s acetate to WT levels (Table 4). In contrast, the *pae9* mutants display an increase in acetate specifically in fraction I by approximately 30 % (Table 4). The *PAE9* overexpression lines in the *pae9*-*1* background show that acetate in this fraction is restored to WT levels (Table 4).

The *pae8/pae9*-*2* double mutant exhibits increased acetate phenotypes in all fractions with the exception of fraction III, which is in agreement with an additive phenotype of the single mutants (Table 4). In the double mutant the rhamnose content is also reduced in fractions II (40 % reduction) and III (13 % reduction) as is the galacturonic acid content in fraction II [(30 % reduction); Tables 2, 3; Fig. S3b, c]. All results are consistent with the proposed pectin acetylesterase activities.

### Recombinant PAE8 and PAE9 release acetate from mutant pectin fractions in vitro

**Fig. 7:**
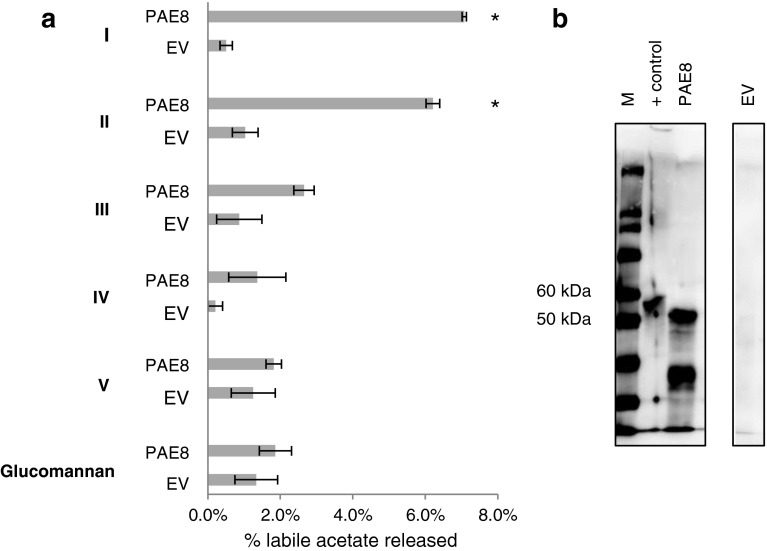
PAE8 releases acetate in vitro from *pae8* pectin fractions. **a** Percentage acetate released from pectin fractions (I–V) and *Amorphophallus konjac* acetylated glucomannan by heterologously expressed PAE8. Alkali would release 100 % of acetate in those fractions. Pectic fractions were generated from three independent *pae8* biological replicates. The same protein content was used for PAE8 and EV activity assays. *Asterisk* indicates significant differences based on *t* test (*P* < 0.015; *n* = 3); *EV* protein extracts purified from tobacco plants transformed with an empty vector; *PAE8* protein extract purified from tobacco plants transformed with PAE8:6XHIS. **b** *Western blot* showing the presence of PAE8 protein (~55 kDa) derived from tobacco plants transformed with the PAE8:6XHIS construct. +control = multi-tag positive control (Life Technologies). *EV* native protein extracts purified from tobacco plants transformed with an empty vector. *M* Magic Marker^TM^ XP (Life Technologies)

**Fig. 8:**
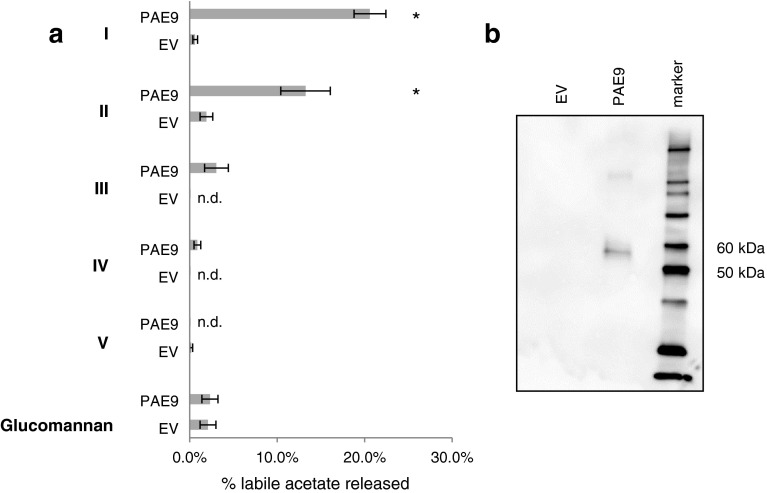
PAE9 releases acetate in vitro from *pae9* pectin fractions. **a** Percentage acetate released from pectin fraction (I–V) and *Amorphophallus konjac* acetylated glucomannan by heterologously expressed PAE9. Alkali would release 100 % of acetate in those fractions. Pectic fractions were generated from three independent *pae9*-*1* biological replicates. The same protein content was used for PAE9 and EV activity assays. *Asterisk* indicates significant differences based on *t* test (*P* < 0.015; *n* = 3); *EV* protein extracts purified from tobacco plants transformed with an empty vector; *PAE9* protein extract purified from tobacco plants transformed with PAE9:6XHIS. **b** *Western blot* showing the presence of PAE9 protein (~55 kDa) derived from tobacco plants transformed with the PAE9:6XHIS construct. *EV* native protein extracts purified from tobacco plants transformed with an empty vector. *Marker* Magic Marker^TM^ XP (Life Technologies)

In vitro activity assays of heterologously expressed proteins were performed to investigate the biochemical activity for PAE8 and PAE9. Both proteins were tagged (6XHis) and transiently expressed in *N. benthamiana* (Figs. 7b, 8b). The chimeric PAE8 and PAE9 proteins were purified using affinity chromatography, and as a control the empty vector (EV) protein extract was processed in the same manner. Both proteins expressed with slightly higher molecular weights (~55 kDa) than the predicted sizes of 45.3 and 46.8 kDa for PAE8 and PAE9, respectively (Figs. 7b, 8b). As expected, no His-tagged proteins were detected in empty vector transformed tobacco (Figs. 7b, 8b).

In vitro activity assays were designed to investigate pectin acetylesterase activity by utilizing the pectin fractions derived from the mutants as substrates (Fig. 6). When PAE8 and PAE9 were incubated with the corresponding mutant pectic fractions, acetate was released from fractions I and II, but not from other fractions (Figs. 7a, 8a; Table S1). PAE9 was able to release more acetate from its corresponding substrate in vitro than PAE8. The observed activities are consistent with the phenotypes of increased acetate found in the pectic fractions I and II of *pae8* and *pae9* mutants (Table 4), but not for *pae8* in fractions IV and V (Table 4).

The transcriptome analysis of the developing corms of *Amorphophallus konjac* revealed the presence of an acetylesterase within the 100 most abundant transcripts that have high protein sequence similarity to PAE8 [VLAE, vodoo lily acetyl esterase; Fig. 1; (Gille et al. 2011a)]. Since *Amorphophallus konjac* contains mainly *O*-acetylated glucomannan as a storage polymer (Chua et al. 2010; Li et al. 2005) this protein was designated as a putative glucomannan *O*-esterase [Fig. 1; (Gille et al. 2011a)]. Hence *O*-acetylated glucomannan was also tested as a substrate for PAE8 and PAE9. However, neither enzyme exhibited any activity against this substrate under the conditions tested (Figs. 7a, 8a).

## Discussion

PAE8 and PAE9 represent pectin acetylesterases based on the in vitro assays with recombinant proteins (Figs. 7a, 8a) and the elevated levels of pectin *O*-acetylation in the corresponding Arabidopsis mutants (Fig. 4b; Table 4). WT and mutant fractions I and II contain mainly RGI (Tables 2, 3; Fig. S3a, b), but the galacturonic acid/rhamnose ratio indicates that HG is also present, consistent with pectin structural models that HG is covalently linked to RGI (Yapo 2011). Elevated acetate levels were observed for high-molecular-weight fractions in all three single mutants (*pae8*, *pae9*-*1* and *pae9*-*2*) indicating that the acetate level of RGI, but also potentially HG, is increased in those mutants. There is no increase in the galacturonic acid content in the pectic fractions of the high acetate mutants (Table 3; Fig. S3; Fig. S4), and the SEC profiles are not altered (Fig. 6) suggesting that the increased acetate content has no effect on HG digestion (Sengkhamparn et al. 2009) and fragmentation pattern. Therefore, RGI seems to be the preferred substrate for PAE8 and PAE9 in planta. Indeed, there is some evidence for reduced extractability of RGI based on the reduction of rhamnose content in the pectin extracts (*pae8* and *pae8*/*pae9*-*2*) and fractions II and III (*pae8*/*pae9*-*2*; Table 2). Less RGI could represent a higher degree of acetylation in this polymer since these mutants show increased acetate levels in the pectin extract and fraction II (Table 4). Highly acetylated wall molecules are more difficult to break-down by enzymes (Busse-Wicher et al. 2014). PAE8 and PAE9 represent acetylesterases with distinct substrate specificities based on the additive effects observed in the double mutant (Fig. 4). The highly complex structure of the RGI molecule entails such diverse substrate sites for these enzymes (Nakamura et al. 2002; Ridley et al. 2001; Yapo 2011).

While the mutants (*pae8*, *pae9*-*1, pae9*-*2* and *pae8/pae9*-*2*) harbor enzymatically extractable pectins with higher acetate contents the remaining residue still contained approximately half of the wall acetate of each line, whose levels were also altered in the mutants (Fig. 4b). The residue still contains pectins based on the monosaccharide compositional analysis (Tables 2, 3; Fig. S2c). However, the residue also contains other wall polymers such as heteroxylan and heteromannan [Fig. S2c; (Pettolino et al. 2012)], which can be *O*-acetylated. Therefore, there is a possibility that PAE8 and PAE9 also act on other acetylated wall polymers. Unfortunately, very few isolated, structurally defined *O*-acetylated wall polymers are available, because the procedures to isolate wall polymers usually entail harsh conditions such as strong alkali (Albersheim et al. 2011), resulting in the removal of acetyl substituents from isolated wall polymers. One of the substrates that is available is *O*-acetylated glucomannan isolated from *Amorphophallus konjac* (Gille et al. 2011a), which, when tested with recombinant PAE8 and PAE9, did not represent a substrate (Figs. 7a, 8a). While both enzymes do not exhibit mannan acetylesterase activity the possibility that they act on other wall polymers than RGI cannot be excluded.

According to the phylogenetic tree of the Arabidopsis pectin acetylesterase family *PAE8* and *PAE9* seem to be the only genes without close paralogs, whereas the other 10 genes clade in 5 pairs (Fig. 1). In Arabidopsis such recent genome duplication events have led to genetic redundancy in some traits (Vision et al. 2000). Hence, PAE8 and PAE9 seem to represent functionally non-redundant proteins. Indeed, the mutant analyses demonstrated that in corresponding mutants of only these two genes a consistent acetate phenotype could be observed as well as an additive acetate increase in the double mutant (Fig. 4a). The generation of double mutants of the other members of the PAE family which clade together and might thus be functionally redundant should prove their substrate specificity.

Some of the first mutants identified to be involved in pectin biosynthesis exhibited dwarf phenotypes, hence named QUASIMODO (Bouton et al. 2002). The *qua1* mutant (putative galacturonic acid transferase) exhibits a shortened inflorescence size and general dwarfed phenotype (Bouton et al. 2002). Another mutant believed to be involved in the methylesterification of homogalacturonan, *qua2*, also shows defects in plant development and growth (Mouille et al. 2007). These findings parallel with our observation that mutants impaired in normal pectin acetylation result in plants with shorter inflorescences (Fig. 5a, b). The growth phenotype identified in this study is manifested in the stem (Fig. 5a, b), which is a tissue that contains only low amounts of pectins when compared to leaves (Xiong et al. 2013). However, it has been reported that pectin biosynthetic genes can be highly expressed in the vasculature (Harholt et al. 2006; Orfila et al. 2005). Pectin structures like galactan are also found in some types of tissues like tension wood which predominantly harbor secondary walls (Arend 2008). These observations suggest that pectin could have an important role in plant tissue development, even in tissues, where it is not highly abundant such as Arabidopsis inflorescence stems. Hence, the inability to detect a wall acetate increase in the mutant stems (Fig. 5c) despite a high expression of *PAE8* and *PAE9* in this tissue does not imply that the reduced stem height is not related to PAE activity.

Plant pectin acetylesterases act on wall polymers after their synthesis and partially remove once added *O*-acetyl substituents (Gou et al. 2012; Orfila et al. 2012). The *pae8/pae9*-*2* double mutant phenotype leads to pectins with a 37 % increase in pectin *O*-acetylation (Fig. 4a) demonstrating the importance of PAEs to modulate the degree of this substituent on pectin. This finding parallels the action of pectin methylesterases (PME) which have been shown to de-methylesterify pectins post synthesis (Micheli 2001). The demethylesterification of HG by PME35 was shown to contribute to the mechanical strength of stem tissues in Arabidopsis (Hongo et al. 2012). It has also been shown that PME genes play a role in pathogen resistance. PME mutants in Arabidopsis promoted enhanced growth of *Pseudomonas syringaea* without altering total plant PME activity, associating specific pectin de-methylation patterns to pathogen resistance (Bethke et al. 2014). In strawberries a direct correlation between wall PME activity and fruit tissue softening has been established (Draye and Van Cutsem 2008). These results highlight the impact of post synthesis modifications on wall biology.

The loss of function of acetylesterase genes in the *pae8/pae9*-*2* double mutant leads to a 37 % increase in total wall acetate levels (Fig. 4a)—a plant biomass feature that has not been achieved previously. The ability to engineer plant biomass with increased acetate content conveys advantageous attributes for biorefinery processes and human health aspects. Increased levels of acetate in hydrolysate from biomass can act as an antimicrobial agent in industrial yeast fermentation processes increasing hygiene and thus overall efficiency (Wei et al. 2013). The use of solventogenic bacteria in fermentation processes of high acetate biomass hydrolysate can help increase solvent formation (Anbarasan et al. 2012). As part of the human diet acetate in indigestible fibers (hemicelluloses and pectin) is released in the colon. Elevated acetate levels in the colon have been associated with the suppression of appetite and obesity control (Frost et al. 2014).

### *Author contributions*

AdeS carried out experiments, analyzed the data and wrote the manuscript; PAH identified PAE8 knockout lines, SG characterized acetate content in pae8; MP conceptualized research, analyzed the data and wrote the manuscript.

## Electronic supplementary material

Below is the link to the electronic supplementary material.

Supplementary material 1 (DOCX 654 kb)
