# Supplementary material for: Identification and functional characterization of the distinct plant pectin esterases PAE8 and PAE9 and their deletion mutants
Source: Planta. 2014 Aug 13;240(5):1123–38. doi: 10.1007/s00425-014-2139-6 (PMC4200376; doi:10.1007/s00425-014-2139-6)
Supplement: Supplementary file 1 — Supplementary material 1 (DOCX 654 kb) [file 425_2014_2139_MOESM1_ESM.docx]

**Titles: Identification and functional characterization of the distinct plant pectin esterases PAE8 and PAE9 and its deletion mutants.**

Amancio de Souza^a^, Philip A. Hull^a,b^, Sascha Gille^a,c^, Markus Pauly^a 1^

^a^ Energy Biosciences Institute, University of California, Berkeley, California 94720, United States.

^b^ Gladstone Institute of Virology and Immunology, PO Box 419100, San Francisco, California 94141-9100, USA

^c^ BayerCropScience, Weed Control Biochemistry & Biotechnology, 65929 Frankfurt am Main, Germany

^1^To whom correspondence should be addressed. E-mail: [mpauly69@berkeley.edu](mailto:mpauly69@berkeley.edu)

Corresponding author: Markus Pauly

Supplementary. Tables

**Suppl. Table S1** Pectin acetylesterase activity

|  | EV- | EV+ | PAE8- | PAE8+ | EV- | EV+ | PAE9- | PAE9+ |
| --- | --- | --- | --- | --- | --- | --- | --- | --- |
| Fraction I | 0.39  ± 0.57 | 0.51  ± 0.17 | 1.09  ± 0.09 | 7.08*  ± 0.06 | n.d. | 0.64  ± 0.28 | 1.21  ± 0.33 | 20.60*  ± 1.82 |
| Fraction II | 0.70  ± 0.22 | 1.03  ± 0.35 | 1.59  ± 0.47 | 6.21*  ± 0.19 | 1.01  ± 0.96 | 1.92  ± 0.73 | 3.48  ± 0.54 | 13.25*  ± 2.82 |
| Fraction III | 0.86  ± 0.24 | 0.87  ± 0.63 | 1.15  ± 0.13 | 2.65  ± 0.28 | 1.15  ± 0.02 | n.d. | 2.40  ± 0.95 | 3.06  ± 1.35 |
| Fraction IV | 1.05  ± 0.05 | 0.21  ± 0.22 | 0.49  ± 0.09 | 1.37  ± 0.79 | n.d. | n.d. | 0.16  ± 0.14 | 0.89  ± 0.39 |
| Fraction V | 1.93  ± 0.31 | 1.25  ± 0.61 | 1.1  ± 0.50 | 1.82  ± 0.21 | n.d. | 0.18  ± 0.16 | 0.72  ± 0.62 | n.d. |
| Glucomanan | 0.72  ± 0.61 | 1.34  ± 0.59 | 1.28  ± 0.85 | 1.86  ± 0.44 | n.d. | 2.11  ± 0.90 | 1.08  ± 0.74 | 2.33  ± 0.92 |

Pectic fractions or konjac glucomannan (acetylated) were incubated with recombinant PAE8:6XHIS (PAE8) or PAE9:6XHIS (PAE9; +), or the tobacco protein extract from empty vector transformed plants (EV+), or their denatured equivalents (-). The released acetate from the various fractions was determined [% of total alkali labile acetate (100 %)]. Pectic fractions were purified from three independent *pae8* and *pae9-1* biological replicates (n=3). n.d. – not detected

± indicate standard deviation

* significant activities compared to the empty vector control (EV+) based on *T* test (*P* < 0.015)

**Suppl. Table S2**

List of primers

| purpose | Primer sequence 5’ – 3’ |
| --- | --- |
| *pae3-1, pae3-2 -* RT-PCR | FW-GCGGCGATATTCTGGCTTTGG |
|  | REV-CCCAGAGACATCCACTGCATCC |
| *pae5-1, pae5-2 -* RT-PCR | FW-ATGGCGATTCCAAGGTTTAGC |
|  | REV-TCATGTGAAATTCATGTTGTAAC |
| *pae6-1, pae6-2 -* RT-PCR | FW-ATGAGGAGCTTGTTGTTGTGG |
|  | REV-TCACTCGAAAATCAGATTGTG |
| *pae7-1 -* RT-PCR | FW-ACTTATCTCCAAAGCGCCGTC |
|  | REV-GGCACGGAAAGTGTCACAATG |
| *pae7-2 -* RT-PCR | FW-TCCACGGATCTGCAAAGAGTC |
|  | REV-TAGGATTACAAGTCGGCGACG |
| *pae8 -* RT-PCR | FW-ATTAGAACTCCTCTGTTCATTC |
|  | REV-CAGCACACAAGACAAGTGCTGAAG |
| *pae9-1 -* RT-PCR | FW-GCGTTAAGTGCATGAGCGATGCTG |
|  | REV-ACCGCGAGAGTTATGGCCTG |
| *pae9-2 -* RT-PCR | FW-TCGATCTAACAGCGGCTATGG |
|  | REV-CCGGTTAGAAGAGCCTTGTGG |
| *pae10-1, pae10-2 -* RT-PCR | FW-GACTGGTTCTTGGGAATGAGG |
|  | REV-TCCTATCACACGGGTATGGAC |
| *pae11-1, pae11-2 -* RT-PCR | FW-ACGCTATACTCTCCGGTTGTTC |
|  | REV-TTCGTCGTCGCGGATCGAAC |
| *pae12-1, pae12-2-* RT-PCR | FW-TCGTTACTGCGATGGCGCTTC |
|  | REV-AATGAGGCATTGGTTACTACC |
| PTB control for RT-PCR | FW-GCGAATGTCTTATTCAGCTCATACTGATC |
|  | REV-CATTGCTGCTGTTGGTATATCAGAGT |
| *pae9-1/ox9* lines Q-RT-PCR | FW-CTGGATTCTTTCTTGACGCAAT |
|  | REV-TCGAGGTTTTTTTGTATACCCTGTAGA |
| *pae8/comp* lines Q-RT-PCR | FW-TCTTGCTACACTCACTGCCAAA |
|  | REV-CAACAGCTTTTGCTATTGTCGTT |
| PTB control for Q-RT-PCR | FW-ACAACAATGATCGATCTAGGGATTATAC |
|  | REV-ATGTGTTTGCCATCTGAGGAT |
| Cloning of PAE9 cds into overexpression vectors | FW-GGGGACAAGTTTGTACAAAAAAGCAGGCTATGAAGACGACGACTCGGCT |
|  | REV-GGGGACCACTTTGTACAAGAAAGCTGGGTTTAAATATCTAGATTTACCA |
| Cloning of PAE8 promoter + genomic sequence | FW-AAAGAGCTCTCAACAATATACCTTTTTGATTAGT |
|  | REV-GGATACAACACTTCGATGATCAGACCCGGGGA |
| Cloning of PAE9cds for protein expression in Tobacco | FW-CTCGAGATGAAGACGACGACTCGG |
|  | REV-GGATCCTCAATGATGATGATGATGATGAATATCTAGATTTACCAA |
| Cloning of PAE8cds for protein expression in Tobacco | FW-TTCATTTCATTTGGAGAGGACACGCTCGAGATGTTCAAGTTGAAGCAATG |
|  | REV-GATCCTCAATGATGATGATGATGATGAATTGGAGGAGCATCTAGAG |
|  | REV-ATCTCATTAAAGCAGGACTCTAGAGGATCCTCAATGATGATGATGATGATG |

Supplementary Figures


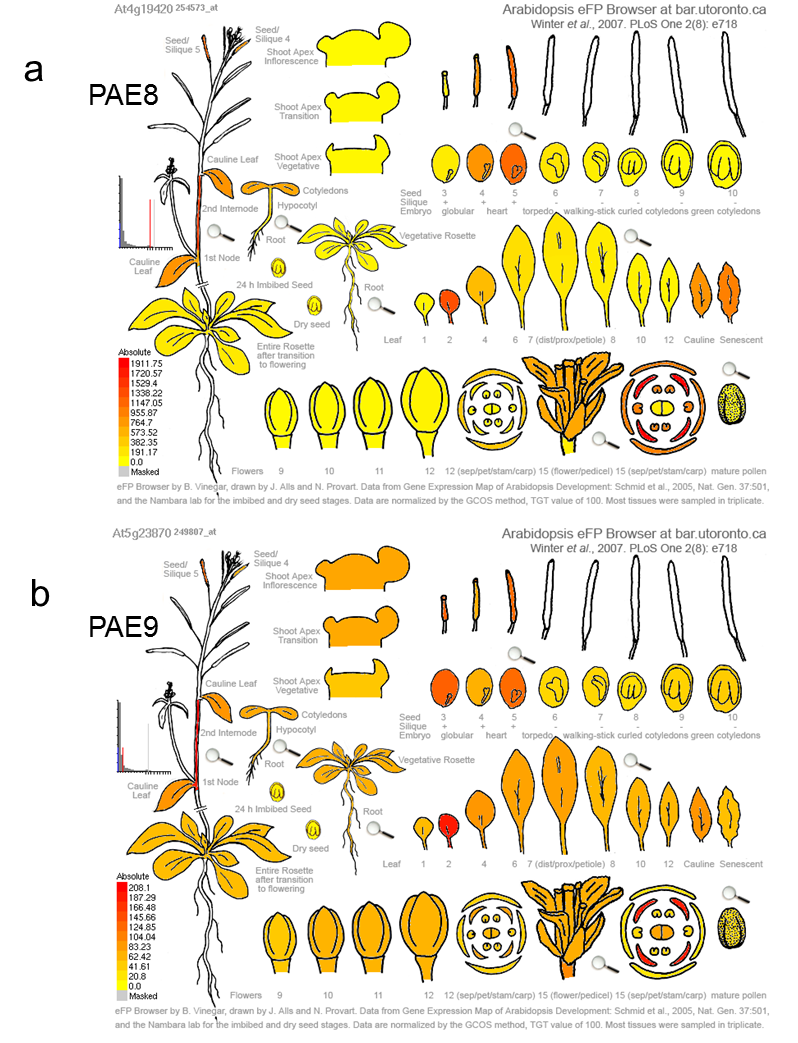


**Suppl. Fig. S1** Expression patterns of **a** *PAE8* and **b** *PAE9*. Gene expression data in *Arabidospsis thaliana* according to the eFP-browser (http://bar.utoronto.ca/efp_arabidopsis/cgi-bin/efpWeb.cgi; Winter et al. 2007). Data represents absolute values of gene expression, scale indicating intensities of expression can be seen on the lower left corner of each section.


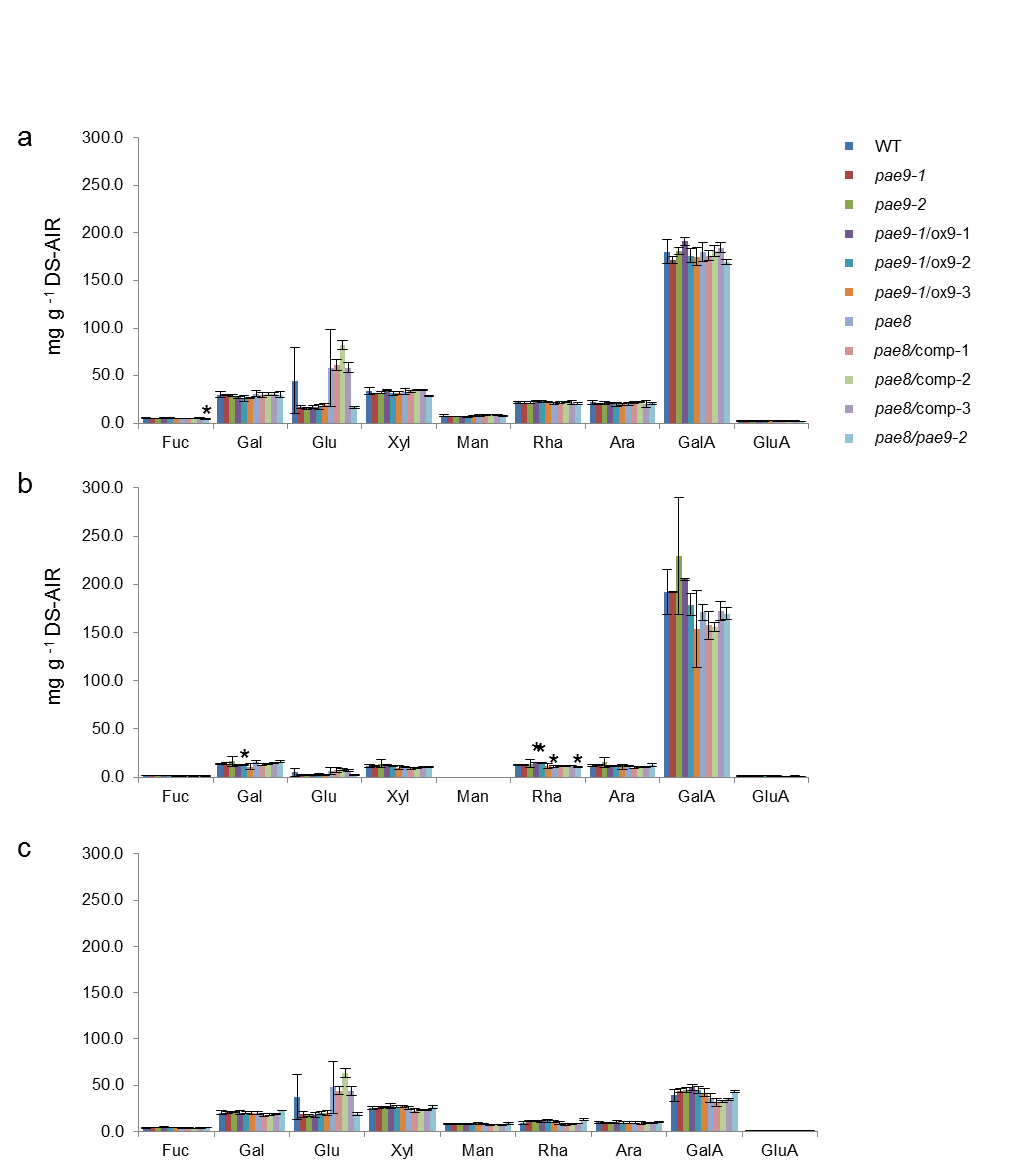


**Suppl. Fig. S2** Monosaccharide composition of cell walls **a**, pectic extract **b** and remaining residue after pectic extraction **c**. * indicates statistical significant differences based on *T* test (*P* < 0.01) , n≥3. ox9 = overexpression of the *PAE9* coding sequence. comp = complementation with the native promoter and genomic sequence of *PAE8*


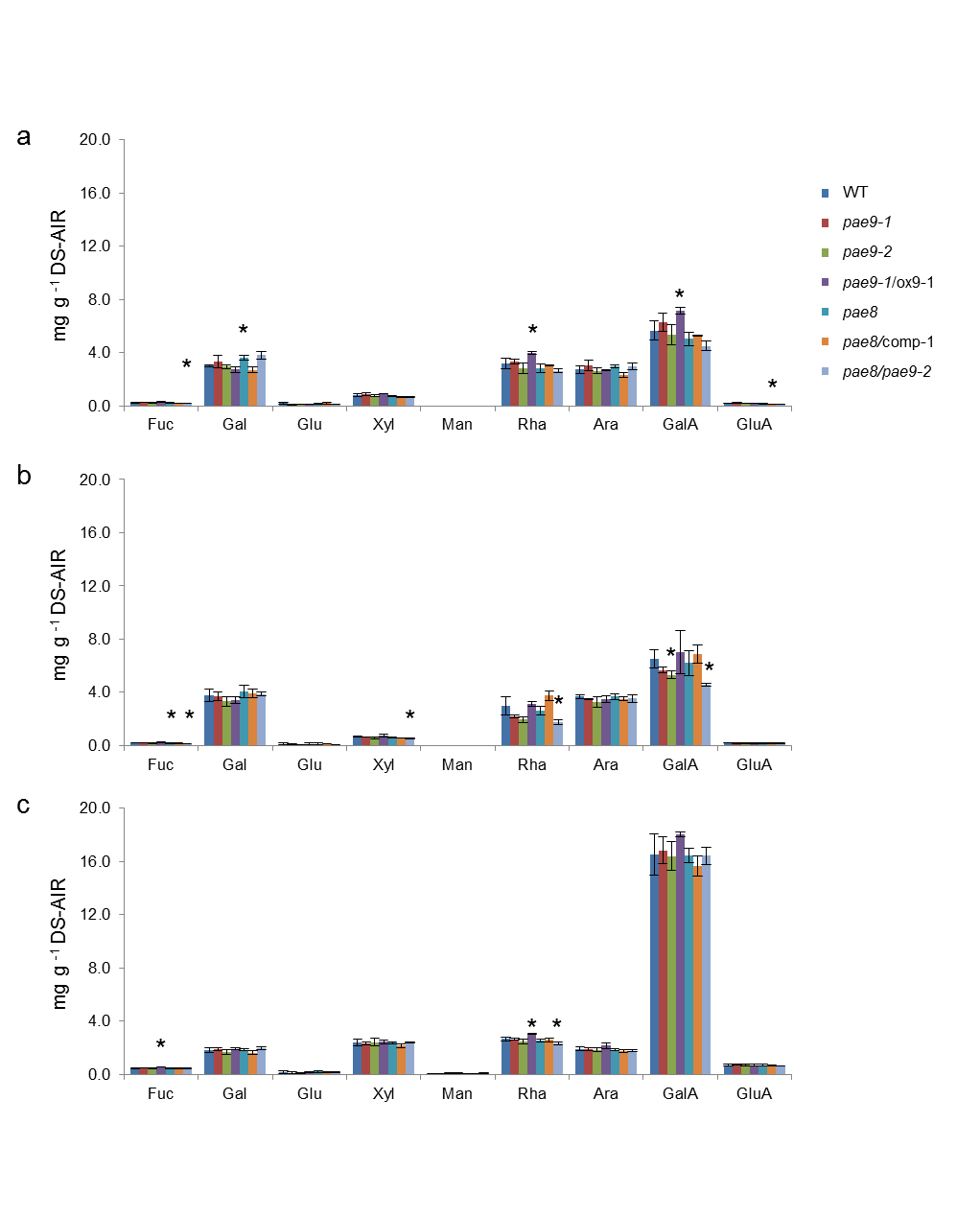
**Suppl. Fig. S3** Monosaccharide composition of pectic fractions I **a**, II **b** and III **c**. * indicates statistical significant differences based on *T* test (*P* < 0.01) , n≥3. ox9 = overexpression of the *PAE9* coding sequence. comp-1 = complementation with the native promoter and genomic sequence of *PAE8*


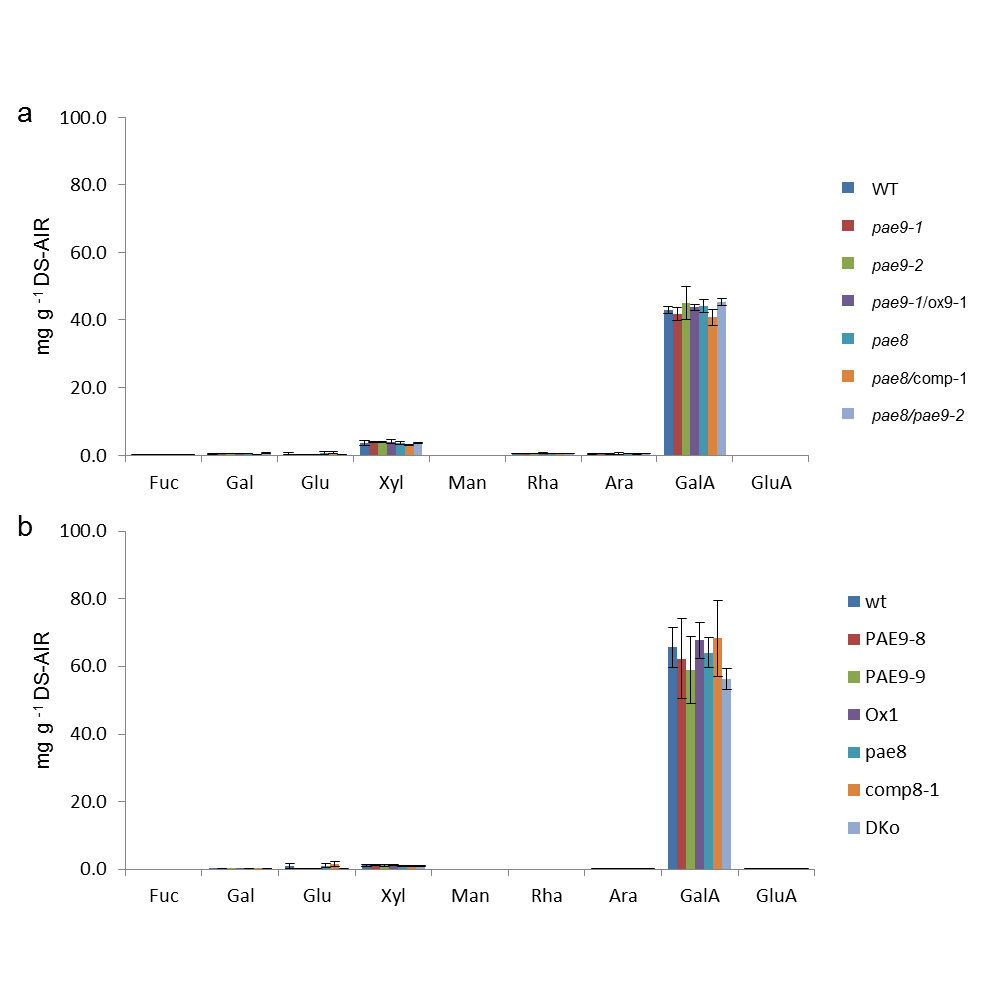


**Suppl. Fig. S4** Monosaccharide composition of pectic fractions IV **a** and V **b**. * indicates statistical significant differences based on *T* test (*P* < 0.01) , n≥3. ox9 = overexpression of the *PAE9* coding sequence. comp-1 = complementation with the native promoter and genomic sequence of *PAE8*
